# A gentler approach to monitor for heart transplant rejection

**DOI:** 10.3389/fcvm.2024.1349376

**Published:** 2024-02-06

**Authors:** Jason F. Goldberg, Aditya Mehta, Rupinder K. Bahniwal, Sean Agbor-Enoh, Palak Shah

**Affiliations:** ^1^ Department of Heart Failure and Transplantation, Inova Heart and Vascular Institute, Falls Church, VA, United States; ^2^ Department of Children's Cardiology, Inova L.J. Murphy Children’s Hospital, Falls Church, VA, United States; ^3^ Eastern Virginia Medical School, Norfolk, VA, United States; ^4^ National Heart, Lung, and Blood Institute (NHLBI), NIH, Bethesda, MD, United States

**Keywords:** acute cellular rejection (ACR), antibody-mediated rejection (AMR), cardiac allograft vasculopathy (CAV), cardiac magnetic resonance (CMR), donor-derived cell-free DNA (dd-cfDNA), donor-specific antibody (DSA), endomyocardial biopsy (EMB), gene expression profiling (GEP)

## Abstract

Despite developments in circulating biomarker and imaging technology in the assessment of cardiovascular disease, the surveillance and diagnosis of heart transplant rejection has continued to rely on histopathologic interpretation of the endomyocardial biopsy. Increasing evidence shows the utility of molecular evaluations, such as donor-specific antibodies and donor-derived cell-free DNA, as well as advanced imaging techniques, such as cardiac magnetic resonance imaging, in the assessment of rejection, resulting in the elimination of many surveillance endomyocardial biopsies. As non-invasive technologies in heart transplant rejection continue to evolve and are incorporated into practice, they may supplant endomyocardial biopsy even when rejection is suspected, allowing for more precise and expeditious rejection therapy. This review describes the current and near-future states for the evaluation of heart transplant rejection, both in the settings of rejection surveillance and rejection diagnosis. As biomarkers of rejection continue to evolve, rejection risk prediction may allow for a more personalized approach to immunosuppression.

## Introduction

The field of heart transplantation has experienced many remarkable innovations including (1) expansion of the donor pool with Hepatitis C-positive donors and donation after circulatory death (DCD) via normothermic regional perfusion and ex vivo perfusion; (2) assessment and understanding of donor-specific antibodies (DSA) and their contribution to antibody-mediated rejection (AMR); and (3) molecular diagnostics including gene expression profiling (GEP), donor-derived cell-free DNA (dd-cfDNA), and DSAs (1–5). Despite these advances, the evaluation of clinical rejection has continued to rely on repetitive invasive procedures, namely the histologic evaluation of endomyocardial biopsy (EMB) specimens to identify and diagnose acute cellular rejection (ACR) and AMR (Table 1). Despite EMB's risk of complications and lack of reproducibility in the diagnosis of rejection, EMB has remained a hallmark of post-transplant care (6, 7).

**Table 1:** Clinically available diagnostics utilized to evaluate heart transplant rejection.

Biomarker	Description	Advantages	Disadvantages
Endomyocardial biopsy (EMB)	Via neck or groin access, a bioptome is used to obtain right ventricular endomyocardial samples	Established grading systems for ACR and AMR	Hospital-based procedure, frequency limited by invasiveness and potential complications, variability of grading across pathologists
Gene expression profiling (GEP)	Transcription evaluation of 11 reporter genes and 9 normalization genes, giving rejection score of 0–40	Can provide reassurance against ACR	Not validated for AMR, cost: ∼$3,000
Donor-derived cell-free DNA (dd-cfDNA)	Evaluation of donor and recipient single-nucleotide polymorphisms (SNPs) to calculate the % dd-cfDNA as the ratio of donor:donor + recipient SNPs.	Higher PPV than GEP, emerging data showing differentiation between ACR and AMR	Cost: ∼$3,000
Donor-specific antibodies (DSAs)	Multiplex bead assays used to evaluate presence of antibodies to donor HLA antigens	Significant association with AMR	Requires serial evaluation to analyze DSA persistence, variability in techniques and reporting across laboratories
Soluble protein biomarkers (troponins and natriuretic peptides)	Assays evaluating circulating, soluble proteins	Readily available in most clinical settings, can signal myocardial injury	Not a sensitive or specific predictor of rejection
microRNA Clinical Rejection Scoring	ACR and AMR scores derived from sequencing of ∼22 base pair non-coding RNA molecules that regulate gene transcription	Ability to identify and distinguish between ACR and AMR	Remains a pre-clinical assay
Cardiac magnetic resonance (CMR) imaging	T1, T2, and ECV CMR sequences to evaluate tissue injury, fibrosis and edema	Provides global myocardial tissue characterization	Limited availability and high cost
Echocardiography	Cardiac ultrasound with traditional 2D as well as deformational (strain) imaging	Can evaluate allograft dysfunction related to rejection	Unable to provide ACR or AMR diagnosis

ACR, acute cellular rejection; AMR, antibody-mediated rejection; PPV, positive predictive value; HLA, human leukocyte antigen; ECV, extra-cellular volume.

**Figure 1:**
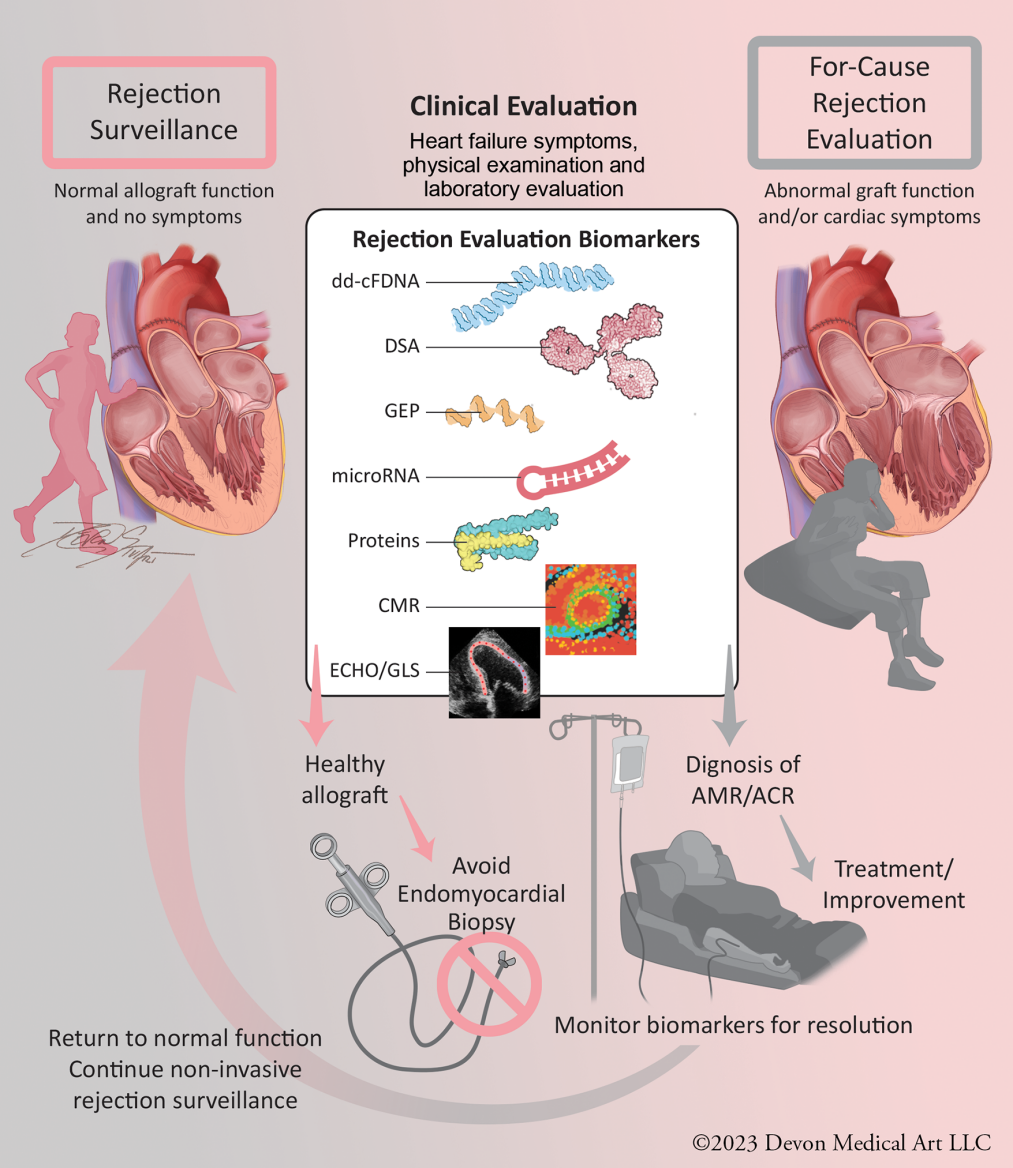
Central figure: evaluation of heart transplant rejection in *surveillance* and *for-cause* settings. Non-invasive molecular and imaging biomarkers can provide clinical reassurance of non-rejection, allowing avoidance of endomyocardial biopsy. With the continued validation of these technologies, they can be used in for-cause rejection evaluation settings to diagnose ACR and AMR and monitor for resolution, allowing clinical improvement and resumption of surveillance status. ACR, acute cellular rejection; AMR, antibody-mediated rejection.

Rejection evaluations occur in two settings (Figure 1): *surveillance* in patients without signs and symptoms of allograft dysfunction (∼98% of EMBs) and *for-cause* in the setting of clinical signs or symptoms of allograft dysfunction (∼2% of EMBs). While transplant centers now forego many surveillance EMBs with reassuring molecular markers, such as dd-cfDNA and/or a lack of DSAs, the high prevalence of abnormal molecular markers in patients without rejection precludes the ability to diagnose and treat patients for rejection based on molecular profiling alone. As these biomarkers are combined with newer rejection evaluation modalities, including cardiac magnetic resonance (CMR) imaging and microRNA profiling, EMB may no longer be necessary to diagnose rejection. The rapid expansion of non-invasive evaluations of heart transplant rejection has resulted in a lack of clear clinical guidance on how to best incorporate them into post-transplant care. This review highlights the molecular and imaging tools available to the clinician in both the *surveillance* and *for-cause* settings. We also present a frontier of *rejection risk prediction*, identifying pre- and post-transplant rejection risk to allow risk stratification and individualization of post-transplant care.

## Rejection surveillance

### Donor-derived cell-free DNA

To date, dd-cfDNA remains the molecular modality with the most robust evidence in the non-invasive identification of both ACR and AMR. The evaluation of cell-free DNA in transplantation stems from preclinical work where donor and recipient plasma whole genome sequencing was performed to identify single nucleotide polymorphisms (SNPs) allowing differentiation and quantification of dd-cfDNA (8). A 2019 publication by Khush et al. describes an initial dd-cfDNA clinical validation: a multicenter, prospective study of 740 heart transplant recipients whose plasma samples were evaluated for dd-cfDNA. A donor:recipient cell-free DNA percentage >0.2% had 54% sensitivity, 76% specificity, PPV of 12%, NPV of 97%, and AUC of 0.64 for identifying a composite of ACR or AMR (Table 2) (9). Another commercially available dd-cfDNA assay utilized a greater number of SNPs in a separate cohort and demonstrated 79% sensitivity, 77% specificity, PPV of 25.1%, NPV of 97.3%, and AUC of 0.86 for the detection of ACR/AMR. Finally, the multicenter Genomic Research Alliance for Transplantation (GRAfT) consortium described results of dd-cfDNA shotgun sequencing, with dd-cfDNA >0.25% having 81% sensitivity, 85% specificity, 20% PPV, 99% NPV, and AUC of 0.92 for diagnosing a composite of ACR or AMR (5).

**Table 2:** Significant publications evaluating donor-derived cell-free DNA and microRNA in the evaluation of heart transplant rejection.

Publication	Study name	Patient population & study design	Description	Outcome
dd-cfDNA				
Khush et al. (9)	D-OAR	740 recipients ≥15 y, multicenter, prospective	Derivation and validation of clinically available (Allosure®) assay with 405 SNPs	dd-cfDNA threshold of 0.2% with 12% PPV and 97% NPV to detect ACR ≥ 2R or pAMR ≥ 1
Richmond et al. (50)	DTRT	101 children, 73 adults, multicenter, prospective	94 SNP dd-cfDNA assay (no longer clinically available)	dd-cfDNA threshold of 0.3% with 85% PPV and 82% NPV to detect ACR ≥ 1R or pAMR ≥ 1
Agbor-Enoh et al. (5)	GRAfT	171 adults, multicenter, prospective	Whole genome, shotgun sequencing (>1 million SNPs)	dd-cfDNA threshold of 0.25% with 20% PPV and 99% NPV; distinct prediction of ACR and AMR
Kim et al. (51)		223 adults, cross-sectional	Clinically available (Prospera^TM^) dd-cfDNA assay with evaluation of >13,000 SNPs	dd-cfDNA threshold of 0.15% with 25% PPV and 97% NPV to detect ACR ≥ 2R or pAMR ≥ 1
MicroRNA				
Duong Van Huyen et al. (42)		4 French centers, 113 patients, Case-control study	Pre-selected evaluation of 14 miR transcripts by RT-PCR, from both EMB and serum samples	Four miRs were differentially expressed in tissue and serum and validated in ACR and AMR as compared to controls
Dewi et al. (52)		6 Canadian centers, 63 patients, Case-control study	Pre-selected miRs evaluated by RT-PCR evaluation in serum	Two miRs were associated with ACR, after controlling for immunosuppressive drug levels, kidney function, and C-reactive protein
Constanso-Conde et al. (53)		1 Spanish center, 66 patients, Prospective Cohort study	Pre-selected evaluation of differential miR expression in ACR by RT-PCR	miR-181a-5p was differentially expressed in ACR ≥ 2R with rise and fall pattern associated with development/treatment of rejection
Shah et al. (43)	GRAfT	5 U.S. centers, 157 patients, Case-control study	Next generation sequencing evaluation of miR expression	Distinct miR scores between 0 and 100 were able to identify and distinguish ACR (32% PPV and 98% NPV) and AMR (37% PPV and 97% NPV

ACR, acute cellular rejection; dd-cfDNA, donor-derived cell-free DNA; miR, microRNA; pAMR, pathologic antibody-mediated rejection; NPV, negative predictive value; SNPs, single nucleotide polymorphisms.

Rejection diagnosis via dd-cfDNA and other molecular markers are evaluated against the histopathologic interpretation of an EMB; however, there remains question about the accuracy of this comparison due to the variability of EMB readings across pathologists (4, 6). Given this concern, the GRAfT investigators analyzed episodes of elevated dd-cfDNA without rejection by EMB. Among the 135 cases classified as “false positive” dd-cfDNA (“false negative” EMB) episodes, 21% were observed in patients exhibiting allograft dysfunction by echocardiography (defined by a decline in left ventricular ejection fraction ≥5%), and 44% of these cases were identified prior to a rejection diagnosis, with AMR being more prevalent than ACR (5). These results suggest that dd-cfDNA is an important biomarker of graft injury and rejection regardless of the EMB results. In addition, the PPV which is calculated in prediction of positive EMB, may actually be substantially higher. Combining dd-cfDNA with DSA, clinical, and imaging evaluations may be sufficient in surveillance of rejection.

### Gene expression profiling (GEP)

The initial biomarker developed to non-invasively identify ACR was GEP, which measures 20 genes (11 reporter genes and 9 house-keeping genes). Individual expression of the 11 reporter genes is quantified and a GEP “score” is calculated with a score >30 (maximum score 40) having a positive predictive value (PPV) of 6.8% and negative predictive value (NPV) of >99% in the evaluation of ACR (4). In the Invasive Monitoring Attenuation through Gene Expression (IMAGE) study patients randomized as early as 6-months post-transplant to a strategy of GEP surveillance vs. EMB-based surveillance had similar outcomes (10). While the inclusion of GEP evaluation into post-transplant care marked the beginning of the “molecular era” of post-transplant evaluation, this biomarker is limited by its inability to detect AMR and lack of reliability when patients are on higher doses of corticosteroids, receive blood transfusions, or have a cytomegalovirus infection (11, 12). In addition, studies evaluating combination testing with GEP and dd-cfDNA show that patients with a positive GEP test and negative dd-cfDNA do not experience rejection, suggesting GEP as a false-positive in this setting (13, 14). As such, GEP is now of historical value in contemporary heart transplant practice, given the availability of dd-cfDNA in resource rich settings.

### Soluble protein biomarkers

The diagnosis of acute cardiac rejection differs from other cardiac conditions, such as acute cardiac ischemia and myocardial infarction, in which a sensitive and specific evaluation can be conducted with high-sensitivity cardiac troponin (hs-cTn) assays and EKG. Both troponin and natriuretic peptides have been extensively studied as predictors of heart transplant rejection, with neither showing reproducible sensitivity and specificity in diagnosing rejection (15–17). Despite hs-cTn greatly improving accuracy over earlier generation troponin assays in ischemic heart disease, a recent evaluation showed no association between hs-cTn and ACR (18). Authors have also queried whether markers of inflammation, such as C-reactive protein and interleukin-6 (IL-6) can diagnose rejection, but neither have proven to be accurate markers (17, 19). There have been recent investigations into proteomic (expression assays of multiple protein biomarkers) evaluations of rejection and graft dysfunction, though rigorous evaluations of proteomic profiling in rejection have yet to be completed (20, 21). While abnormal troponins and/or natriuretic peptides can signal allograft dysfunction related to rejection, their lack of significant evidence in diagnosing rejection limits their use in rejection surveillance.

### Rejection surveillance without biopsy

Many heart transplant centers now forego routine surveillance biopsy as early as one month after transplant in patients with favorable molecular evaluations (13). One single-center study evaluated this practice in 153 recipients where reassuring dd-cfDNA results allowed the cancellation of 84% of surveillance biopsies (14). This evaluation included a protocol recommending EMB cancellation with dd-cfDNA <0.2%. Of 172 biopsies performed during the one-year study period, only 2 patients had ACR and 2 patients had AMR: all episodes of rejection occurred in the setting of dd-cfDNA >0.2%. In another single-center evaluation, 64 recipients underwent 475 dd-cfDNA evaluations within the first year post-transplant, with molecular evaluation prompting EMB in only 22 cases (with 2 biopsies yielding ACR), while clinical evaluation led to EMB in 3 cases (with 1 biopsy yielding ACR) (13). As many centers are eliminating surveillance EMB with reassuring molecular and clinical evaluations, there are multiple ongoing trials assessing the effectiveness of these non-invasive rejection surveillance strategies.

## Rejection diagnosis

Within five years of heart transplantation, over 40% of recipients are hospitalized for acute rejection, and repeat episodes of rejection increase the risk of graft dysfunction and cardiac allograft vasculopathy (CAV, a manifestation of chronic rejection), the leading cause of mortality in the late phase post-transplant (22, 23). The relationship between the patient and the transplant team involves frequent monitoring for signs and symptoms of rejection, which include fatigue, decreased appetite, dyspnea, edema, and weight gain. These symptoms would prompt an in-person assessment with clinical evaluation to include physical examination, echocardiography, and laboratory assessment of end-organ function and cardiac biomarkers (troponins or natriuretic peptides). Despite a clinical picture that suggests rejection, these medical and laboratory evaluations remain non-specific in the diagnosis of rejection, and patients are typically referred for a diagnostic EMB, which is evaluated for ACR and AMR based on the International Society for Heart and Lung Transplantation (ISHLT) grading nomenclature (24, 25). Increasingly, these for-cause evaluations include molecular testing such as DSAs and/or dd-cfDNA, which may provide rejection diagnosis without EMB. As these evaluations and other emerging biomarkers gain clinical utility, it may be possible to diagnose and treat rejection without EMB.

### Donor-Specific antibodies (DSAs)

Immunologists and transplant physicians have worked together to advance the field of *immuno-evaluation*, where antibody data is used to assess humoral immune system activity before and after transplant. Given the limited donor pool, patient acuity, and in contrast to kidney transplantation, heart transplantation does not rely on human leukocyte antigen (HLA) matching. Rather, recipients are evaluated to ensure limited cross reactivity between pre-formed HLA antibodies and donor HLA antigens at the time of a donor offer through a process known as virtual crossmatch. As the complexity of heart transplant recipients increases (including mechanical circulatory support use, congenital heart disease, and multiparous females), heart transplant candidates may carry significant levels of pre-formed HLA antibodies, reducing the number of potential donors and increasing the risk of DSA development post-transplantation. DSA development post-transplant is associated with increased risk for AMR, CAV, allograft dysfunction, and mortality (26–29). In the past, evaluation of HLA antibodies (both pre-formed to the potential donor pool and post-transplant, specific to the donor organ—DSA) required physical complement dependent cytotoxicity (CDC) crossmatching where donor cells and recipient serum were combined to assess cross reactivity and cell lysis. This has evolved into virtual assessments of cross reactivity using solid-phase immunoassays (SPIs) where recipient serum is incubated with multiplex bead assays containing HLA antigens (30, 31).

Specific DSA characteristics, including their persistence throughout multiple post-transplant evaluations as well as their specificity to Class II HLA (primarily at the HLA-DQ locus), have been associated with higher rates of rejection and graft failure (32, 33). While there is a significant association between DSA and AMR, DSA is also found in ACR, reflecting the interdependence between the cellular and antibody-mediated immune response in rejection (27, 29). Currently, DSA evaluation alone cannot be used as a surrogate of rejection, as many patients develop DSA but never have rejection. Rather, it is used in complement with clinical and other molecular modalities to signal potential rejection. As more is learned about DSA development and associated risk factors, its evaluation will continue to be important in the evaluation of rejection.

### Imaging evaluations

As CMR tissue characterization has aided in the evaluation of post-ischemic myocardial viability and inflammatory cardiomyopathies, there have now been numerous evaluations of its use in rejection diagnosis. Multiple CMR sequences including T1 and T2 mapping and extracellular volume (ECV) have been associated with acute rejection. In an evaluation of 112 simultaneous EMBs and CMRs, Imran et al. showed that native T1 mapping was able to identify a composite of ACR/AMR, with 93% sensitivity, 79% specificity, 99% NPV, and AUC of 0.89 (34). In another study by Dolan et al. of 72 subjects, global and peak T2 signals had the highest values in patients with active ACR and had sequentially lower signals in those with prior ACR followed by further decreased signal among healthy controls (35). This study also showed global and segmental ECV to be significantly higher in patients with active ACR, regardless of presence or absence of past ACR. Recent results of a randomized, non-inferiority trial of EMB vs. CMR rejection surveillance in 40 heart transplant recipients in Sydney, Australia showed 92% sensitivity, 93% specificity, 62% PPV, 92% NPV, and AUC 0.92 in the detection of composite ACR/AMR (36). In the CMR group, study protocol allowed for escalation to EMB in equivocal or clinically determined settings, which was only necessary in 6% of cases. Comparing the EMB and CMR surveillance groups, rejection rates were similar and the rates of hospitalization and infection were lower in the CMR group. Wider use of CMR for rejection diagnosis will require larger scale clinical trials as well as prompt availability of CMR in rejection suspicion (similar to rapid MRI evaluation utilized in stroke protocols). In addition, the cost of CMR varies greatly across the globe, which may limit universal availability.

Traditional 2D echocardiography can signal allograft dysfunction with the development of systolic or diastolic dysfunction. There remains question, however, about the utility of advanced echocardiography techniques, such as strain imaging, in the evaluation of rejection. Assessment of 2D global longitudinal strain (GLS) imaging has been associated with ACR, including a 2010 analysis by Kato et al. of 396 simultaneous EMB and GLS evaluations, with GLS > −27.4% having 82% sensitivity, 82% specificity, 36% PPV, and 97% NPV for ACR (37). While these results have been replicated in other studies, there have also been studies showing a lack of association between GLS and rejection (38). Additionally, there remains concern that cardiac strain imaging may not be consistently reproducible in the clinical setting, limiting its application in rejection evaluation (39). As advanced imaging modalities continue to evolve, they may complement clinical and molecular investigations of rejection, allowing non-invasive diagnosis of rejection.

### Diagnosing rejection without biopsy

**Figure 2:**
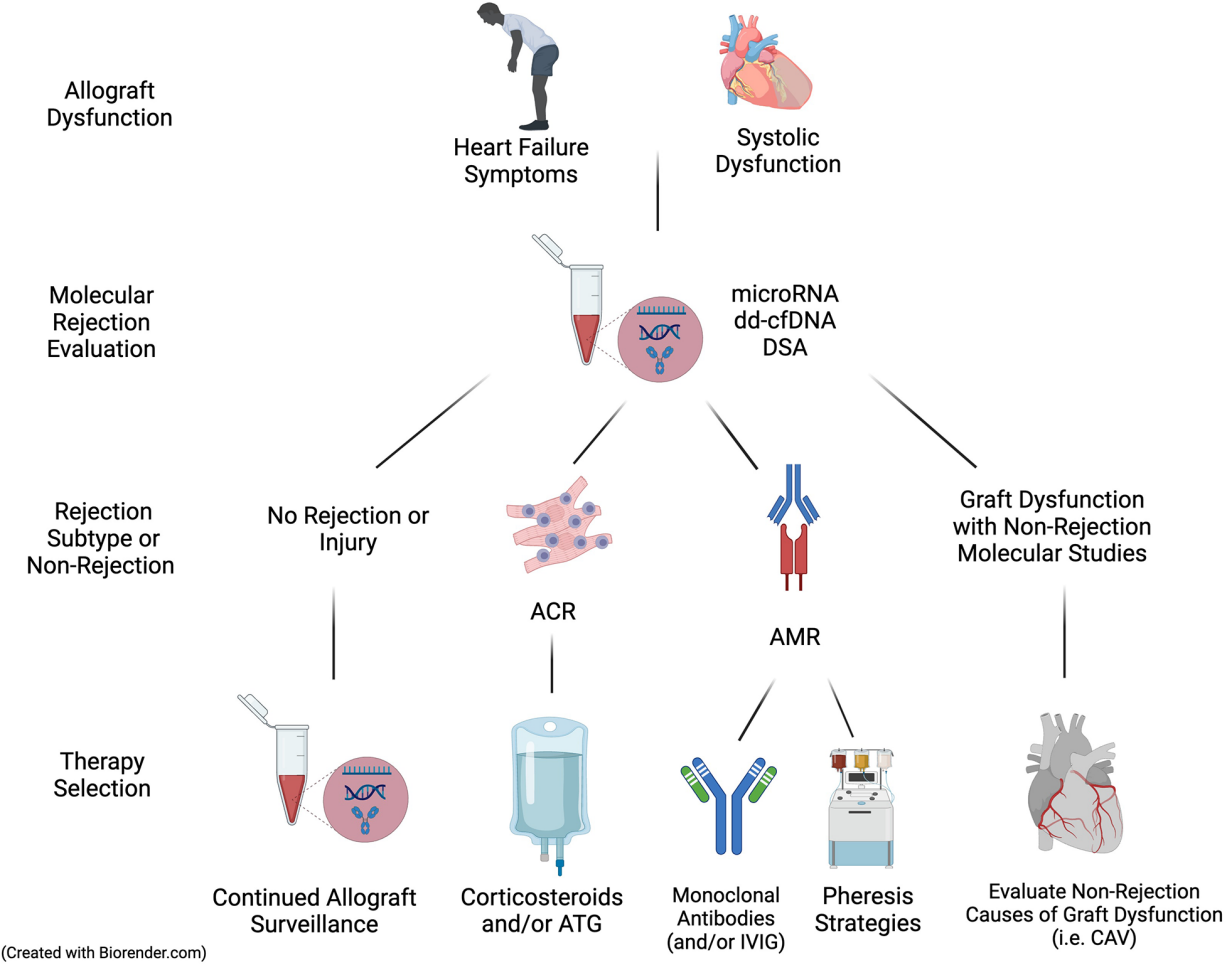
Utilizing molecular evaluation to diagnose and treat acute cellular rejection and antibody-mediated rejection. With continued clinical validation, molecular biomarkers may allow the diagnosis of specific rejection subtype or support addition evaluations, allowing appropriate therapy without the need for EMB. ACR, acute cellular rejection; AMR, antibody-mediated rejection; ATG, anti-thymocyte globulin; CAV, coronary artery vasculopathy; dd-cfDNA, donor-derived cell free DNA; DSA, donor-specific antibody.

With the addition of positive molecular biomarkers of rejection to a clinical picture of allograft dysfunction (such as decreased ejection fraction or signs of heart failure), standard practice has been to initiate pulse dose corticosteroids and proceed with EMB to permit histopathologic assessment for ACR and/or AMR. This includes traditional hematoxylin and eosin staining which can provide an ACR diagnosis as well as special immunohistochemical stains to facilitate the diagnosis of AMR. If ACR is identified, most patients are treated with corticosteroid therapy and, in those with graft dysfunction, anti-thymocyte globulin may be administered. The diagnosis and treatment of AMR, however, is more complex, highlighting the need for an accurate diagnosis to outline a precise therapeutic pathway. Therapies for AMR include monoclonal antibodies, intravenous immunoglobulin, and apheresis strategies that impart substantial infection risk and/or cost. The suspicion for AMR is raised in recipients with pre-transplant HLA antibody sensitization, *de novo* post-transplant DSA, prior AMR, or those of Black race (27, 40, 41). With the presence of these factors as well as circulating DSA and/or other molecular markers of AMR, the non-invasive diagnosis of AMR may be possible (Figure 2).

Recent publications of molecular biomarkers suggest the ability to potentially discriminate between ACR and AMR without an EMB. Agbor-Enoh and Shah et al. demonstrated that dd-cfDNA has comparable diagnostic performance in assessing ACR (AUC 0.89) and AMR (AUC 0.95) individually (5). These results from the GRAfT consortium also showed distinct dd-cfDNA characteristics between rejection subtypes, with AMR having significantly higher dd-cfDNA values, shorter dd-cfDNA fragment lengths, and a higher percentage of guanosine:cytosine bases. Emerging modalities, including that of *microRNAs* may further allow for the diagnosis of rejection subtype without EMB. Duong van Huyen et al. showed that microRNAs were differentially expressed in ACR as compared to AMR in both the myocardium and circulation of heart transplant patients with rejection (42). More recently, a GRAfT study developed and validated microRNA clinical rejection scores for both ACR and AMR, having AUCs of 0.86 and 0.84, respectively (43).

Catheterization and EMB carry risk, especially in the setting of rejection, where these procedures can precipitate hemodynamic compromise. They also are resource-dependent, which may delay the initiation of rejection therapy or lead to non-specific therapy. As molecular and imaging biomarkers of rejection continue to evolve in a diagnosis-specific manner, EMB may not be needed to initiate rejection therapy. There remains the entity of non-specific graft failure or biopsy-negative rejection, a diagnosis which may be further elucidated and adequately treated with molecular biomarker differentiation of ACR or AMR phenotype.

## Rejection risk prediction

It has become clear that a universal approach to post-transplant care may be less favorable than a personalized medicine approach frequently weighing each patient's rejection risk, which is dynamic as a function of time post-transplant. The 2022 ISHLT Guidelines for the Care of Heart Transplant Recipients support dd-cfDNA and DSA monitoring, without a recommendation for the elimination of EMB with reassuring evaluations (44). They include a Class IIa, Level of Evidence C recommendation to perform DSA monitoring at 1, 3, 6, and 12 months, followed by annually thereafter as well as more frequent assessments for sensitized patients. However, there are no specific schedules recommended for dd-cfDNA monitoring, citing a lack of significant evidence. The incorporation of clinical characteristics and non-invasive biomarkers may lead to multi-item risk prediction modeling that can be readily and repeatedly calculated for each individual patient, in a fashion similar to the AHA/ACC Atherosclerotic Cardiovascular Disease (ASCVD) Risk Calculator. In the long-term evaluation of CAV in heart transplant recipients, Loupy et al. described trajectory-based modeling to identify multivariable risk for CAV (45). Risk modeling of rejection, however, would require real-time rejection prediction, combining post-transplant biomarkers including DSA and dd-cfDNA with known pre-transplant predictors of rejection including HLA antibody sensitization and demographic factors. A composite risk score could help guide rejection monitoring, prompting more or less frequent evaluations given specific patient risk.

Prediction of rejection risk should also include immune system assessment, to help guide immunosuppressive therapy. Current immunosuppression strategies include induction (rapid immune state suppression to induce transplant tolerance) at time of transplant followed by lifelong maintenance immunosuppression with monitoring of circulating immunosuppression medication levels. Medications are typically tapered in a non-precise manner, decreasing doses and desired medication levels at set time intervals after transplant. In addition, levels are often reduced when a patient develops drug toxicity such as gastrointestinal intolerance, leukopenia, renal insufficiency, or neurologic dysfunction. Drug level and toxicity monitoring is important, as differences in pharmacogenomic and physiologic states require altered immunosuppression doses. However, ideal immune monitoring would include an evaluation of net state of immunosuppression, determining risk for rejection with under-immunosuppression and risk for infection or malignancy with over-immunosuppression. The Immuknow® Assay [Viracor-IBT (formerly Cylex), Lenexa, Kansas] has been used toward this goal; however, clinical evaluations of this assay have not shown a significant association with rejection (46, 47). Rather, real-time molecular evaluations such as dd-cfDNA (measuring donor allograft quiescence), torque teno virus quantification (measuring net immunosuppression load), and microRNA (measuring epigenetic factors regulating immune system transcription) may provide insight into immune state (5, 48, 49). These biomarkers have the potential to predict risk of not only rejection (with under-immunosuppression) but also complications of over-immunosuppression (such as infection and malignancy). As these modalities continue to be studied, a rejection risk prediction model may provide a comprehensive evaluation of each patient’s risk profile and the impact of specific rejection therapy and monitoring strategies.

## Conclusion

At many heart transplant centers, patients undergo a gentler approach to rejection surveillance, with the elimination of many routine *surveillance* EMBs in the setting of favorable clinical, imaging, and/or molecular evaluations. As more data emerge regarding the positive predictive value of molecular and advanced imaging evaluations of ACR and AMR, many *for-cause* biopsies may also be eliminated. This shift may enable non-invasive rejection diagnosis and prompt initiation of rejection therapy. Molecular biomarkers continue to transform the way physicians treat patients, potentially creating *rejection risk prediction* tools for heart transplant recipients, allowing for less invasive evaluation and improved post-transplant longevity.
